# GPCRs in hypothalamic neurons and their roles in controlling food intake and metabolism

**DOI:** 10.3389/fnmol.2025.1536577

**Published:** 2025-02-05

**Authors:** Tian Qiu, Ou Fu

**Affiliations:** ^1^School of Biotechnology, Jiangnan University, Wuxi, Jiangsu, China; ^2^Laboratory of Food Perception Science, Science Center for Future Foods, Jiangnan University, Wuxi, Jiangsu, China

**Keywords:** hypothalamus, GPCR (G protein coupled receptor), GLP-1 receptor, melanocortin receptor, NPY receptor, food intake, metabolism

## Abstract

G-protein coupled receptor (GPCR) subtypes within the hypothalamus play a pivotal role in maintaining body homeostasis, particularly in the regulation of food intake and energy metabolism. This review provides an overview of classical loss and gain-of-function studies on GPCRs related to feeding and metabolism, with a focus on emerging cell-type-specific investigations. These studies reveal that diverse GPCR-expressing neuronal populations are intricately linked to feeding and energy balance. We also discuss recent findings that highlight the interaction of distinct peptide-GPCR systems in modulating complex feeding behaviors.

## Introduction

The global rise in obesity and associated metabolic disorders, largely due to unhealthy lifestyles and excessive consumption of energy-dense foods, presents a significant health challenge (Simpson et al., 2015; NCD Risk Factor Collaboration (NCD-RisC), 2016; Baak, 2023; Lingvay, 2024). Addressing this issue requires a comprehensive understanding of the central mechanisms that drive overeating (Schwartz et al., 2000; Rossi and Stuber, 2018; Zimmerman and Knight, 2020). G protein-coupled receptors (GPCRs), a vast family of seven-transmembrane receptor proteins (Hilger et al., 2019), have emerged as pivotal targets for numerous pharmacological interventions for metabolic diseases, including obesity and diabetes (Liu et al., 2024).

The hypothalamus, a critical brain region essential for the regulation of feeding and metabolism (Coll et al., 2007; Brüning and Fenselau, 2023), is enriched with GPCRs related to these functions (Sobrino Crespo et al., 2014; Krashes et al., 2016; Kabahizi et al., 2022). Recent advancements in GPCR drug development have shown promise in treating obesity. For example, semaglutide, a glucagon-like peptide-1 receptor (GLP-1R) agonist, has demonstrated reliable weight loss effects in treating obesity and diabetes (Knudsen and Lau, 2019; Davies et al., 2021). Similarly, melanocortin-4 receptor (MC4R) agonist, setmelanotide, is effective for the treatment of genetic forms of obesity (Clément et al., 2020; Markham, 2021; Clément et al., 2018; Haws et al., 2020). Expanding our knowledge of GPCR functions in the hypothalamus is fundamental for the future improvement of GPCR drugs for metabolic syndrome.

In this review, we focus on key GPCRs and their associated neurons within the hypothalamus that are crucial for feeding and metabolism, including GLP-1 receptor, melanocortin receptor, neuropeptide Y receptor, corticotropin-releasing hormone receptor, and oxytocin receptor. By presenting the current understanding of the complex roles and interactions between peptide-GPCR systems in the hypothalamus, we aim to offer new insights into the future development of anti-obesity medications.

## GLP-1 receptors

Glucagon-like peptide-1 receptors (GLP-1Rs) have become a key target in obesity treatment, garnering significant attention. Semaglutide and Liraglutide, developed as long-acting GLP-1R agonists, have shown dramatic weight loss effects in treating metabolic disorders, including obesity and type 2 diabetes (Knudsen and Lau, 2019). Moreover, a meta-analysis study demonstrates that administration of GLP-1 receptor agonists in diabetes patients exhibits slightly better effects on blood glucose control compared with insulin therapy (Abd El Aziz et al., 2016).

In central, GLP-1Rs are heavily expressed in the hypothalamus, including the arcuate nucleus (ARC), paraventricular nucleus of hypothalamus (PVH), and dorsomedial hypothalamus (DMH; Gu et al., 2013; Cork et al., 2015). Electrophysiology study has shown that the application of GLP-1R agonist exendin-4, significantly enhances the action potential (AP) firing rate of ARC^GLP-1R^ neurons. Furthermore, activation of these neurons, performed by the excitatory DREADD hM3Dq, results in a notable suppression of food intake without significant changes in blood glucose or insulin levels (Singh, 2022). GLP-1R neurons in the PVH receive projections from the NTS, activation of glucagon (Gcg) neurons in the NTS inhibits feeding behaviors, while deletion of PVH^GLP-1R^ neurons increases food intake and decreases locomotor activities, leading to obesity (Liu et al., 2017). In another study, it is reported that the activity of PVH^GLP-1R^ neurons increase temporarily during refeeding and chemogenetic activation of these neurons also significantly reduce appetite (Li et al., 2019).

**Figure 1:**
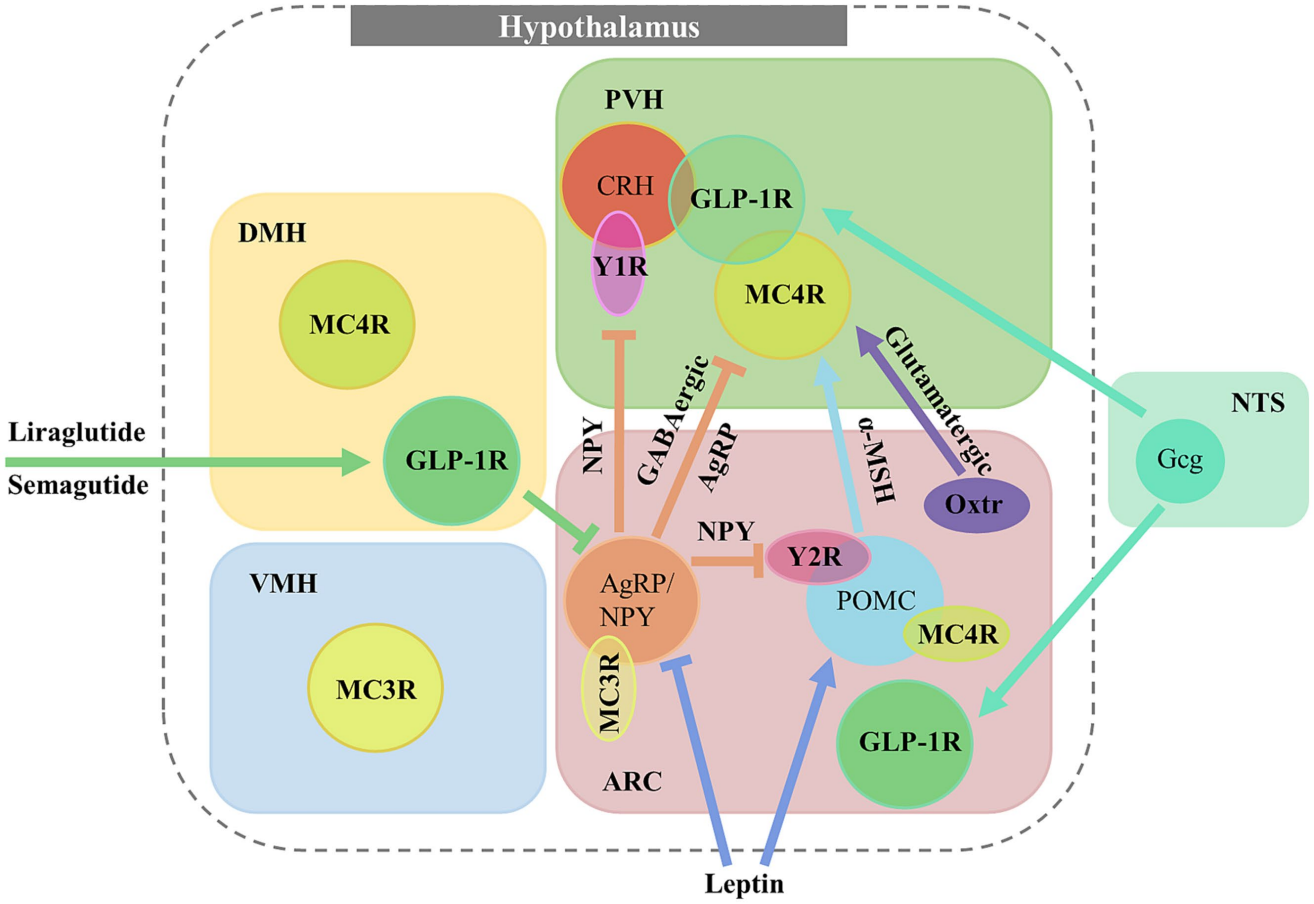
GPCR expression in hypothalamus and neural networks for regulating feeding and metabolism. GLP-1R and MC4R are differentially expressed in numerous hypothalamic areas, distributed in PVH, DMH, VMH or ARC. GLP-1R neurons in PVH and ARC are activated by endogenous GLP-1 from NTS^Gcg^ neurons while DMH^GLP-1R^ neurons are activated by GLP-1R agonist drugs (Liraglutide and Semagutide). Leptin signaling activates POMC neurons but inhibits in AgRP neurons. PVH^MC4R^ neurons are activated by *α*-MSH from POMC neurons and inhibited by AgRP neurons. In the ARC, AgRP neurons inhibit NPY2R in POMC neurons and NPY1R on PVH^CRH^ neurons by release of NPY.

GLP-1Rs in the DMH also play a crucial role in metabolism and are suggested to be a major target for GLP-1R agonist drugs (Figure 1). Chemogenetic activation of GLP-1R neurons in the DMH, innervated by NTS^Gcg^ neurons, reduces blood glucose levels, whereas ablation of GLP-1R in the DMH elevates them, suggesting that the endogenous GLP-1 system is crucial for maintaining blood glucose balance (Huang et al., 2022). Recent findings also implicate DMH^GLP-1R^ neurons are involved in pre-ingestive satiation (Kim et al., 2024), indicated by an increased self-reported satiation index in human after semaglutide administration. Optogenetic inhibition of DMH^GLP-1R^ neurons reduces feeding motivation in fasted mice, while activation of the same neurons increases appetite. Furthermore, *in vivo* calcium imaging reveals that a subset of DMH^GLP-1R^ neurons is tuned to response during food seeking, supporting the hypothesis that these neurons encode the pre-ingestive satiation. In addition, ARC^AgRP^ neurons are identified as the downstream target of DMH^GLP-1R^ neurons to elicit satiation. Collectively, these observations strengthened the reliability of GLP-1R agonist-based drug therapies for obesity.

## Melanocortin receptors

Melanocortin receptors (MCRs), particularly the melanocortin-4 receptor (MC4R), are crucial GPCR in regulating physiological metabolic processes (Sweeney et al., 2023). The recent approval of Setmelanotide, an MC4R agonist, for treating obesity caused by deficiencies in proopiomelanocortin (POMC), proprotein convertase subtilisin/kexin type 1 (PCSK1), or Leptin Receptor (Markham, 2021), has heightened scientific interest in the effects of MCRs on feeding and energy balance (Table 1).

**Table 1:** Cell-specific manipulations of GPCR expressing neurons and their impact on food intake and metabolism.

Nucleus	GPCR (neuron)	Genetic or neural manipulations	Food intake and metabolism
ARC	MC4R (POMC)	Deletion (*POMC-Cre: MC4Rflox/flox*)	Energy expenditure↓ (Guo et al., 2024)
	MC3R (AgRP)	Deletion (*AgRP-Cre:MC3R-flox*)	Food intake↓ (In cold and fasting state; Gui et al., 2023)
	MC3R	Chemogenetic activation (*MC3R-Cre with AAV-hM3Dq*)	Food intake↑ (Sweeney et al., 2022)
	GLP-1R	Chemogenetic activation (*GLP-1R-Cre with AAV- hM3Dq*)	Food intake↓ (Singh, 2022)
	NPY2R (POMC)	Deletion (*Pomc^Cre/+^: Npy2r^lox/lox^*)	Food intake↓ (Qi, 2023)
	NPY2R (NPY)	Deletion (*Y2^lox/lox^: NPY^cre/+^ with tamoxifen inducing*)	Food intake (High fat diet) ↑ (Qi et al., 2016)
	OXTR	Optogenetic activation (*Oxtr-cre with AAV-ChR2*)	Food intake↓ (Fenselau et al., 2017)
DMH	GLP-1R	Chemogenetic activation (*GLP-1R-cre wih AAV- hM3Dq*)	Food intake↓ (Kim et al., 2024)
	MC4R	Deletion (DMHGs KO)	Energy expenditure↓ (Chen et al., 2019)
PVH	GLP-1R	Deletion (*GLP-1R^f/f^ with AAV-Cre*)	Food intake↑ (Li et al., 2019)
	NPY1R	Chemogenetic inhibition (*NPY1R-Cre with AAV-hM4Di*)	Glucose and lipid metabolism↓ (Chen et al., 2023)
	MC4R	Optogenentic activation (*MC4R-Cre with AAV-hM3Dq*)	Food intake↓ (Garfield et al., 2015)
		Deletion (*Mc4r^lox/lox^ with AAV-Cre*)	Food intake↑ (Shah et al., 2014)

Among numerous MCR from type1 to type5, MC3R and MC4R are expressed in the brain (Dib et al., 2017), with significant dense distributions in the hypothalamus (Catania, 2010). Both receptors play a pivotal role in feeding and energy homeostasis (Anderson et al., 2016; Butler et al., 2000). MC3R deficient mice show increased fat mass, reduced lean mass and higher feed efficiency than wild-type mice (Chen et al., 2000). MC3R knockout mice display an obese phenotype, restoring MC3R signaling in ventromedial nucleus of the hypothalamus (VMH) Steroidogenic Factor-1(SF-1) neurons, which are involved in the regulation of blood glucose and fat metabolism (Coutinho et al., 2017; Rashid et al., 2023), results in a partial rescue by attenuating changes in fat mass and markedly improving metabolic homeostasis (Begriche et al., 2011). Furthermore, VMH^MC3R^ neurons are sensitive to glucose changes, and their activation promotes glucose disposal (Sutton et al., 2021). Besides the VMH, MC3Rs are also expressed in ARC^AgRP^ neurons (Sweeney et al., 2023), and MC3R signals are required for the activation of AgRP neurons by fasting. Food intake during refeeding following overnight fasting is significantly lower in MC3R knockout mice compared to normal mice, with a decline in c-Fos expression in AgRP neurons (Gui et al., 2023). Another study indicates that MC3R knockout mice exhibit enhanced anorexia. Chemogenetic activation of AgRP^MC3R^ neurons increases feeding, while MC3R specific antagonist C11 inhibits food intake (Sweeney et al., 2022), suggesting that MC3R in the ARC is a potential pharmacological target for anorexia.

MC4R is primarily expressed in the CNS in a distinct set of nuclei from MC3R, predominantly in the PVH (Sweeney et al., 2023). Notably, MC3R is expressed pre-synaptically in AgRP neurons, from which it acts to regulate the release of GABA onto PVH^MC4R^ neurons (Ghamari-Langroudi et al., 2018). The absence of MC4R in PVH leads to hyperphagia (Shah et al., 2014), energy imbalance, increased fat mass, and glucose disorders (Krashes et al., 2016). Electrophysiological studies show significantly increased activity of PVH^MC4R^ neurons when exposed to *α*-MSH, an MC4R agonist from POMC neurons (Fenselau et al., 2017). In contrast, optogenetic activation of ARC^AgRP^ neurons that project to PVH^MC4R^ increases feeding, while chemogenetic activation of the neural pathway from PVH^MC4R^ to lateral parabrachial nucleus (LPBN) significantly reduces food intake (Garfield et al., 2015; Figure 1). Besides, chemogenetic inhibition of PVH^MC4R^ neurons leads to increased food intake in energy-sufficient states (Sayar-Atasoy et al., 2023), indicating that PVH^MC4R^ neurons encode satiety. α-MSH released from POMC neurons can persistently elevates cAMP level in PVH^MC4R^ neurons, which in turn induces satiety and decreases food intake during feeding (Zhang, 2024). Specific ablation of MC4R in ARC^POMC^ neurons also leads to obesity, decreased energy expenditure, and impaired insulin sensitivity. Kir2.1, an inwardly rectifying potassium channel, mediates MC4R function in the ARC^POMC^ neurons. Knockdown of Kir2.1 in MC4R-deficient mice partially restores energy balance and insulin sensitivity (Guo et al., 2024). The DMH^MC4R^ neurons also connect to brown adipose tissue (BAT) via rostral raphe pallidus (rRPa) in brainstem, and its sympathetic innervation generates thermogenesis. Disruption of MC4R or Gαs signaling in the DMH impairs basal and cold-stimulated SNS outflow to BAT, leading to reduced thermogenesis and energy expenditure (Chen et al., 2019).

## NPY receptors

The neuropeptide Y receptors (NPYRs) are a family of GPCRs that respond to the neuropeptide Y, peptide YY, and pancreatic polypeptide. Centrally, NPY receptors are extensively distributed in the hypothalamus, with NPY1, NPY2, and NPY5 receptors being the primary binding targets of endogenous NPY and implicated in the regulation of food intake (Balasubramaniam, 2002; Mercer et al., 2011; Parker and Balasubramaniam, 2008).

In ARC, NPY1R and NPY2R are expressed in both AgRP and POMC neurons, and it is reported that POMC neurons could receive central NPY from AgRP neurons (Cowley et al., 2001). In slice patch clamp study, NPY2R agonists inhibit NPY release from ARC^NPY^ neurons (King et al., 1999). Moreover, the mRNA expression of NPY2R in AgRP neurons is elevated in fasted mice, while no significant difference is observed for NPY1R. On the contrary, when mice were exposed to a high-fat diet, both NPY1R and 2R mRNA levels decreased in POMC neurons and loss of NPY2R in POMC neurons significantly reduces food intake (Qi, 2023). Previous studies suggest that specific NPY2R deletion in postnatal onset NPY neurons did not significantly affect spontaneous food intake but increase fasting-induced food intake (Qi et al., 2016). NPY1R deletion slightly induces increasing in body temperature and significantly increasing body weight but no significantly change in food intake (Kushi et al., 1998). Another recent study indicates that NPY in AgRP neurons modulates food intake via NPY1R and energy expenditure via the NPY2R pathway. In an AgRP neuron-specific NPY deletion mouse model, re-introduction of NPY1R signaling by selective NPY1R agonists increases feeding and Respiratory Quotient (RQ), while NPY2R selective agonist administration elevates energy expenditure and locomotion (Qi et al., 2022).

In addition to ARC, NPY receptors are also expressed in PVH (Fetissov et al., 2004). Early immunostaining studies suggest that some PVH neurons expressing NPY1R receive projections from AgRP neurons (Broberger et al., 1999). This finding has been further substantiated by the Calcium and RNA multiplexed Activity analysis (CaRMA) technique, which confirms the expression of NPY1R in PVH neurons and highlights the strong correlation between their activity and feeding behavior as well as physiological state (Xu et al., 2020). Activation of ARC^AgRP^-PVH pathway has been shown to increase food intake in mice, and blocking NPY1R with a selective antagonist significantly reduces food intake (Atasoy et al., 2012).

## CRH and OXT receptors

Neuroendocrine systems are essential for regulating body homeostasis, including blood pressure, blood glucose level, metabolism. Here, we introduce the CRH-CRHR (Corticotropin-releasing Factor Receptor) and Oxytocin-OXTR (Oxytocin Receptor) system within the PVH based on current findings for their role in regulating food intake.

CRH neurons are enriched in the PVH and function as the apex of the HPA axis, known for the stress response (Swaab et al., 2005). Corticotropin Releasing hormone (CRH) is released from these neurons, binds to CRH receptors on the pituitary gland (Daniel, 1976), triggers ACTH release, and eventually increase blood corticosterone level, a process suggested to alter feeding behaviors (Adam and Epel, 2007; Qi et al., 2020; Jiang and Tong, 2022). Recent *in vivo* calcium imaging for CRH neuron confirmed that these neurons are sensitive to stressors, and their activities could be suppressed by food reward (Yuan et al., 2019; Kim et al., 2019), suggesting that the CRH-CRHR system may function as a hub for regulating feeding behaviors under stress. Moreover, activation of PVH^CRH^ neurons or AMPK modulates macronutrient preference in mice (Okamoto et al., 2018). Disrupted PVH^CRH^ neuron responsiveness by clamping at high or low levels similarly contributes to diet-induced obesity (Zhu et al., 2020).

CRH receptors have two main subtypes: CRHR1 and CRHR2 (Aguilera et al., 2004). CRHR1 is associated with stress perception and is the primary subtype in the pituitary that receives CRH from the PVH (Ramot et al., 2017). Acute stress increases c-Fos expression in CRHR1 positive cells in the PVH, and chronic stress causes an attenuation in the gene expression of CRH1R (Bonaz and Rivest, 1998). Although CRHR1 KO mice do not show change in total food intake but exhibit a change in circadian rhythm, increase oxygen consumption and promote resistance to diet-induced obesity (Sakamoto et al., 2013). CRHR2 in the hypothalamus is also suggested to regulate feeding in different ways under acute or chronic stress (Qi et al., 2020). Following acute stress, CRH binding to CRHR2 reduces NPY expression while increases POMC expression, inhibit feeding.

OXT receptors in the hypothalamus are closely linked with learning, stress, and social behavior (Liu et al., 2023; Osakada et al., 2024), and recent research highlights their roles in feeding behavior and energy metabolism (Kerem and Lawson, 2021). Intracerebroventricular oxytocin activates LepR in hypothalamus, reducing food intake, especially in males (Liu et al., 2020). The paraventricular nucleus (PVH) houses many OXT and OXTR neurons, and their interactions are crucial for the regulating of feeding and body weight (Maejima et al., 2024). Notably, Oxytocin are also reported to regulate fat accumulation, OXTR-deficient mice show an increase of white adipose Tissue (WAT) and a decrease of body temperature compared with controls (Takayanagi et al., 2008), implying that the absence of OXTR might increase the likelihood for overweight and obesity.

OXTR are potential targets for anti-obesity drugs (Niu et al., 2021; Kerem and Lawson, 2021). Peripheral oxytocin binds to receptors on vagus nerve endings (Jurek and Neumann, 2018), signaling to the NTS in the brainstem, which then activates the oxytocin system in the PVH to suppress appetite. Optogenetic activation of ARC^OXTR^-PVH terminals significantly reduces food intake and produces satiety (Fenselau et al., 2017). However, OXTR does not appear to have a significant effect on energy expenditure, as there is no significant alteration in energy expenditure and RQ in male OXTR knockout mice (Kasahara et al., 2013).

## Interaction within distinct peptide-GPCR systems and other appetite regulating modules in the hypothalamus

We have discussed numerous peptide-GPCR systems and their functions in the regulation of feeding and metabolism (Table 1). These appetite-related GPCRs are often expressed in AgRP neurons and POMC neurons, which are known to encode opposing internal states (Van De Wall et al., 2008), representing hunger and satiety. Moreover, genes encoding different peptides or GPCRs are also co-expressed in the same neuron populations in the hypothalamus, identified by immunohistology, *in situ* hybridization, or more recent single cell RNA-seq analysis (Steuernagel et al., 2022; Figure 1). These properties enable potential interactions between distinct peptide-GPCR systems and other appetite regulating modules.

PVH is considered as an important nucleus for such interaction as it contains a plenty of neuropeptides and GPCR expressing neural populations. Previous anterograde tracing study suggests that CRH neurons that express NPY1R in PVH serve as a direct downstream target of NPY neurons in ARC (Li et al., 2000). Recent RNAscope data indicates a considerable overlap of GLP-1R, MC4R, CRH and OXT expressing in PVH, particularly MC4R, CRH, and OXT neurons show high levels of GLP-1R expression (Li et al., 2019), implying that these neurons might receive regulation from central GLP-1. Another study shows that NPY released from AgRP neurons in ARC acts on PVH^NPY1R^ neurons, which in turn activate adjacent PVH^CRH^ neurons to enhance of lipid and glucose metabolism (Chen et al., 2023). In addition, CRHRs are expressed in a proportion of oxytocin neurons in PVH (Ugartemendia et al., 2022), suggesting that CRH and oxytocin system may constitute a reciprocal regulation for stress-induced changes in feeding.

Peptide-GPCR interactions also exist in another important appetite regulating module, known as the leptin and its receptor, LepR (Patterson et al., 2011; Friedman, 2019; Rossi, 2023). Leptin released from peripheral adipocytes could across the blood–brain barrier and function on LepR expressing hypothalamic neurons to regulate energy metabolism (Allison and Myers, 2014; Butiaeva et al., 2021; Duquenne et al., 2022). In the lateral hypothalamic area (LH), MC3R neurons modulate locomotor activity, energy expenditure, and adiposity, and a proportion of these neurons overlap with LH^LepR^ neurons, suggesting that MC3R neurons might receive leptin signals to synergistically modulate energy balance (Pei et al., 2019). In addition, single-cell RNA sequencing and fluorescent ISH data indicate that there are a large number of neurons co-express GLP-1R and LepR in DMH and ARC (Rupp et al., 2023). Loss of the leptin receptors in LepR^GLP-1R^ neurons provoke hyperphagic obesity without impairing energy expenditure. In contrast, restoration of GLP-1R expression in LepR^GLP-1R^ neurons in GLP-1R-null mice enable inhibition of food intake by the GLP-1R agonist, liraglutide. These data suggest that melanocortin, leptin and GLP-1 system may collaborate to regulate feeding and energy homeostasis through specific neural populations.

Besides the direct interactions within neuropeptide and GPCR co-expressing neurons, probably via specific neuronal populations or neural circuits, leptin signals in ARC could also indirectly influence MC4R functions in the PVH, which is described as a part of central leptin–melanocortin pathway. Leptin activates POMC but inhibits AgRP neurons (Friedman, 2019), thus modulates the function of their downstream target PVH^MC4R^ neurons. Furthermore, leptin administration upregulates POMC gene expression and downregulates AgRP and NPY gene expression (Casanueva and Dieguez, 1999; Seoane-Collazo et al., 2020). In addition, it is reported that diminished AgRP signals to the PVH^MC4R^ neurons and elevated *α*-MSH from POMC neurons reduce food intake and enhance satiety (Deem et al., 2022).

## Conclusion and perspectives

In the hypothalamus, GPCR-expressing neurons regulate feeding behavior and energy homeostasis in many aspects. Distinct peptide-GPCR systems are not only operated independently in the brain, these signals are also integrated, which in turn modulate complex feeding behaviors in distinct internal states. Understanding these mechanisms is beneficial for developing novel therapeutic drugs and strategies for obesity induced metabolic disorders.
